# Development of the Canadian Eating Practices Screener for Adolescents to assess eating practices based on Canada’s Food Guide 2019 recommendations

**DOI:** 10.1186/s12966-025-01853-1

**Published:** 2025-12-16

**Authors:** Raphaëlle Jacob, Marciane Any, Virginie Desgreniers, Geneviève Bessette, Rita Al Kazzi, Alicia E. Martin, Claire Tugault-Lafleur, Kimberley Hernandez, Sylvie St-Pierre, Jess Haines

**Affiliations:** 1 Department of Family Relations and Applied Nutrition, University of Guelph, 50, Stone Rd East, Guelph, Ontario N1G 2W1, Guelph, Canada; 2 School of Nutrition, Faculty of Health Sciences, University of Ottawa, Ottawa, Canada; 3 Department of Geography, Environment and Geomatics, University of Guelph, Guelph, Canada; 4 Food and Nutrition Directorate, Health Canada, Ottawa, Canada

**Keywords:** Eating practices, Questionnaire, Validation, Content validity, Cognitive interviewing, Dietary guidelines, Youth, Adolescents, Children, Canada

## Abstract

**Background:**

In addition to guidance on food choices, the Canada’s Food Guide 2019 (CFG-2019) provides recommendations to support healthy eating habits. A brief self-administered eating practices questionnaire informed by CFG-2019 recommendations was recently developed and validated among adults, but no such measure is available for adolescents. The objective of this study was to develop and assess the content validity of a self-administered screener to measure eating practices based on CFG-2019 recommendations among English- and French-speaking adolescents aged 10 to 17 years.

**Methods:**

Following a literature review of existing measures and the identification of guiding principles for questionnaire development, a 26-item draft screener was created. The content validity of the draft screener was assessed by an expert panel with expertise in nutrition, eating behaviours, public health and/or questionnaire validation (English *n* = 13, French *n* = 7) and through two rounds of cognitive interviews with adolescents (English *n* = 18, French *n* = 13).

**Results:**

The number of items was reduced from 26 to 12 following review by the expert panel, and further reduced to 11 after the cognitive interviews with adolescents. Minor wording changes were made to improve clarity of a few items.

**Conclusions:**

This study resulted in the development of the 11-item Canadian Eating Practices Screener for Adolescents/Questionnaire court canadien sur les pratiques alimentaires des adolescents designed for use among adolescents aged 10 to 17 years. Further work is needed to test the screener for construct validity and reliability. After which, this measure can be used for research and nutrition surveillance of eating practices among adolescents living in Canada.

**Supplementary Information:**

The online version contains supplementary material available at 10.1186/s12966-025-01853-1.

## Background

As stated in the 2019 version of Canada’s Food Guide (CFG-2019): “*Healthy eating is more than the foods you eat. It is also about where*,* when*,* why and how you eat.”* [1]. For the first time since its inception, the 2019 version of the CFG provides recommendations on eating practices, or eating habits, in addition to guidance on food choices [1]. These recommendations on eating practices are being mindful of what you eat – which includes taking time to eat and noticing hunger and satiety cues – enjoying your food, eating meals with others, cooking more often, using food labels, and being aware that food marketing can influence food choices. The CFG-2019 and the accompanying dietary guidelines aimed to promote healthy eating and overall nutritional well-being of Canadians aged two years and older, and support improvements to the food environments [2].

Food guides are tools developed by many countries to translate national dietary guidelines into simple and practical recommendations to help the population achieve dietary patterns associated with overall health and chronic disease prevention [2, 3]. They are also used to guide policy and program development [2, 3]. Consequently, for surveillance and research purposes, it is essential to assess the population’s dietary behaviours recommended in these guidelines [3]. A series of instruments have recently been developed and validated to assess dietary intake and eating practices recommended in CFG-2019 [4–9]. These instruments include brief questionnaires, also called screeners, designed to rapidly assess dietary intake and eating practices based on CFG-2019 among adults [6–9]. Brief instruments are practical for epidemiological research and surveillance purposes given their lower participant burden, especially among youth and in contexts where several questionnaires are concurrently administered [10–12]. However, such tools are not currently available to assess dietary intakes and eating practices recommended in CFG-2019 among children and adolescents living in Canada.

Adolescence is an important developmental period for establishing long-term eating habits. Research has shown that eating practices established during this life stage, such as participating in food preparation and eating family meals, persist into adulthood, and predict higher diet quality in both adolescence and adulthood [13–20]. Moreover, learning to cook at a young age, compared with learning during adulthood, is associated with higher food literacy and diet quality in adulthood [14]. Similarly, consuming an unhealthy diet and engaging in unhealthy behaviours, such as eating while watching TV, during adolescence have also been shown to persist into adulthood [18, 21, 22]. These findings highlight adolescence as a key period for developing healthy eating practices. Given that adolescents have lower numeracy and literacy levels than adults, and experience different roles and responsibilities around eating, tools uniquely designed for this age group are needed to support research and surveillance efforts during this life stage [23]. With regard to eating practices, some existing questionnaires validated for use with children and adolescents comprehensively assess some of the eating practices recommendations outlined in CFG-2019 (e.g., Adult Eating Behaviour Questionnaire validated among adolescents [24, 25], Mindful Eating Questionnaire for children [26], Childhood Family Mealtime Questionnaire [27, 28]), but no questionnaire briefly assesses all of the CFG-2019 recommendations on eating practices among adolescents.

This study aimed to develop the Canadian Eating Practices Screener for Adolescents/Questionnaire court canadien sur les pratiques alimentaires des adolescents, which assesses eating practices outlined in CFG-2019 recommendations on healthy eating habits, for use with adolescents aged 10–17 years. This paper describes the development process of the screener, including the assessment of content validity among experts and adolescents [29–33]. A separate brief questionnaire, i.e., the Canadian Food Intake Screener for Adolescents/Questionnaire court canadien sur les apports alimentaires des adolescents, assessing food intake based on CFG-2019 recommendations on healthy food choices, is described in the accompanying paper [34].

## Methods

**Fig. 1:**
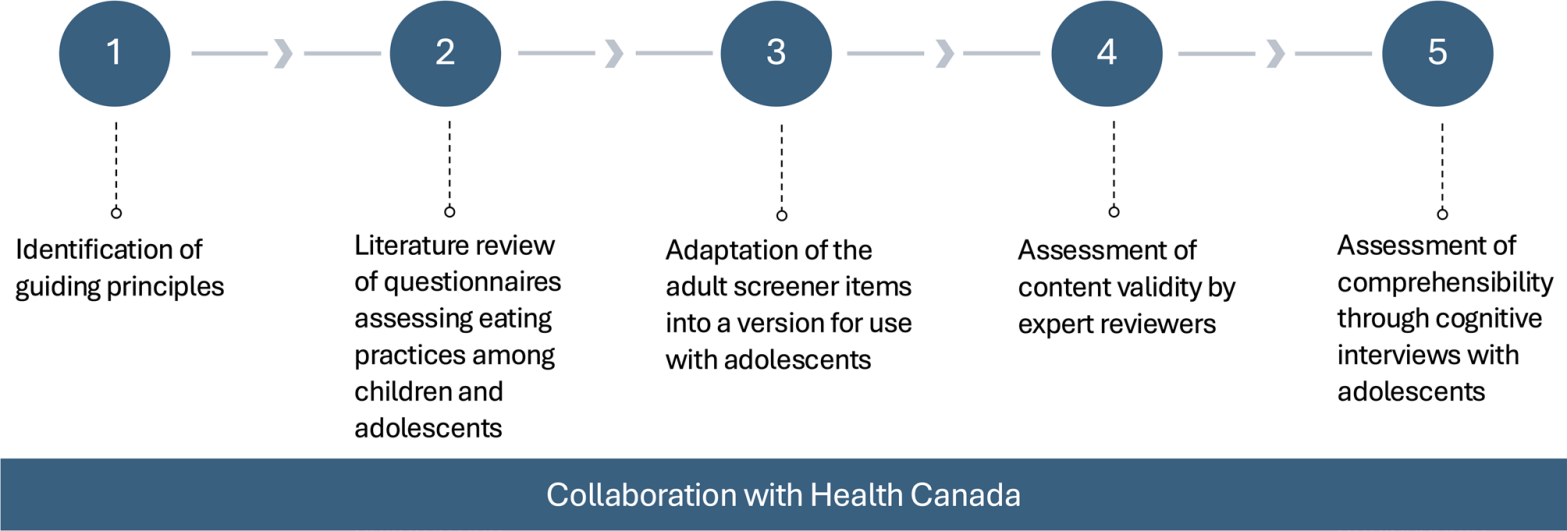
Development process of the Canadian Eating Practices Screener for Adolescents

The Canadian Eating Practice Screener for Adolescents was developed through a five-stage process and ongoing collaboration with Health Canada (Fig. 1). The first step involved the establishment of guiding principles for developing the screener. The second step consisted of a review of the literature on questionnaires assessing similar constructs to the eating practices outlined in the CFG-2019 among children and adolescents, followed by the adaptation of the Canadian Eating Practices Screener developed for adults for use with adolescents (Step 3) [8, 9]. Steps four and five involved the assessment of content validity of the English and French versions of the screener through an expert panel and cognitive interviews with adolescents aged 10–17 years, respectively. This five-stage process is consistent with guidelines on questionnaire development from the COSMIN (COnsensus-based Standards for the selection of health Measurement INstruments) initiative [32, 33]. A similar process, followed by the assessment of construct validity and internal consistency, was used to develop the Canadian Eating Practice Screener for use with adults aged 18–65 years [8, 9]. The adult screener was developed and validated in 2021, and includes 21 items [8, 9].

### Step 1: Identification of guiding principles

The guiding principles used to inform the development of the adolescent eating practices screener were based on those used for the development of the adult screener [9] and adapted to reflect the developmental stages of adolescents. They define the constructs to measure, the target population, and the format, considerations, and expectations for the tool. These guiding principles specify that the eating practices screener should assess alignment with the CFG-2019 recommendations on healthy eating habits, be simple to administer and to complete for adolescents aged 10–17 years, be brief (i.e., take less than 5 min to complete), consider the cognitive demand, and the numeracy and literacy levels of the target population, be easily understood by the target population as determined through cognitive interviewing, and be sensitive to the needs and life stage of adolescents, using language that is sensitive and relevant, for example to social inequalities and disordered eating. The screener should also demonstrate adequate construct validity and reliability, which would need to be determined in a subsequent study that specifically examine these aspects.

### Step 2: Literature review of existing questionnaires used with children and adolescents

To inform the adaptation of the adult screener for use with adolescents, Health Canada conducted a literature review of existing tools that have been used to assess the eating practices outlined in CFG-2019, or similar constructs, among children and adolescents. The search was performed in May 2023 and included the literature published since 2010. A total of 637 records were screened for eligibility, among which 21 articles were included in the qualitative review, resulting in the identification of 8 questionnaires (data not shown). Examples of the questionnaires identified through the literature review include the Child Three-Factor Eating Questionnaire [35] which has been validated among English- and French-speaking Canadian children and adolescents aged 8–15 years [36, 37], the Adult Eating Behaviour Questionnaire validated for use in adolescents aged 11–18 years [24, 25], the Mindful Eating Questionnaire adapted for children aged 8–11 years [26], and the Knowledge and Food Practices Questionnaire, which assesses alignment with the Brazilian Food Guide recommendations in children aged 7–11 years [38].

### Step 3: Generation of items included in the Canadian eating practices screener for adolescents

The review of existing questionnaires assessing eating practices in children and adolescents enabled the adaptation of the items, instructions and response scales of the adult screener so that a context- and language-appropriate version could be created for use with adolescents (Supplemental Table 1) . In contrast to the adult screener, which requests information about eating practices over the past month [8, 9], the adolescent screener inquires about eating practices in a more comprehensive manner, using a normal week as the reference point. All items have been adapted for use with adolescents, except the two items assessing the cook more often – involve others in planning and preparing meals recommendation from the adult screener (i.e., *I plan meals with others*, and *I cook meals with others*). For seven items of the adult screener, two items using different wording have been created for adolescents for each adult item, resulting in a total of 26 drafted items for the adolescent screener. For example, the item “*I cook meals from scratch. This means meals that include at least three basic ingredients. Examples of basic ingredients include canned beans*,* meats*,* vegetables*,* rice*,* spices*, etc.” from the adult screener was initially adapted into two items for use with adolescents, namely “*In a normal week*,* how often do you help prepare a meal?”* and “*In a normal week*,* how often do you cook a meal on your own?*". Once adapted for use with adolescents, the screener was translated in French by a nutrition expert and reviewed by another expert in the field. Revisions were made based on a consensus between them. Both experts were native French speakers. When necessary, an additional French-speaking nutrition expert was consulted. The translated version was then shared with bilingual members of the research team for further review. Supplemental Table 1 shows the final items included in the adolescent eating practices screener and the corresponding item from the adult eating practices screener [8, 9].

### Steps 4 & 5: Assessment of content validity with experts and adolescents

Content validity represents the extent to which a questionnaire adequately reflects the construct(s) to be measured in terms of relevance, comprehensiveness, and comprehensibility of the items, response scales and instructions [29–33]. These aspects of content validity were tested through an expert panel (Step 4), while comprehensibility was further tested through cognitive interviewing with adolescents (Step 5) [29, 33].

#### Expert panel

A total of 20 experts were invited and agreed to assess the content validity of the English (*n* = 13 experts) and French (*n* = 7 experts) versions of the screener in January 2024 (Supplementary File S1). These experts were from the government and academic sectors and were invited based on their expertise in nutrition, eating behaviours, public health and/or questionnaire validation. Experts assessed each item for relevance, i.e., whether the item assessed the intended recommendation, and clarity on a binary scale (yes/no). Experts were also invited to provide suggestions for improvements to each item, and to comment on whether important concepts or dimensions of CFG-2019 recommendations on healthy eating habits were missing. Items that were rated as being relevant and/or clear by 75% or fewer experts were removed or modified. Percentages of expert agreement were calculated separately for the English- and French-speaking expert panels, but results and expert comments from both languages were considered together in the decision to remove, modify or retain each item. Given the total number of experts and that results from both languages were considered jointly, a threshold of greater than 75% was used to assess expert agreement on item relevance and clarity [39]. Written feedback was also considered in the decision to remove or modify an item. Consequently, an item that was rated as relevant and clear by more than 75% of experts could also have been removed or modified, for example, depending on whether the item was seen as appropriate for adolescents or to better reflect the corresponding recommendation. Decisions regarding the removal or modification of items were made by a committee consisting of members of the research team and advisors from Health Canada.

#### Cognitive interviewing with adolescents

Comprehensibility of items included in the screener following the expert panel was further tested among English- and French-speaking adolescents aged 10–17 years using cognitive interviewing [40, 41]. Cognitive interviewing is an evidence-based qualitative method designed to investigate whether items of a questionnaire are interpreted and answered as intended by members of the target population [41].

##### Participants

English-speaking participants were recruited through community organizations serving youth and families, word of mouth and an existing research study involving families in Guelph, Ontario, Canada [42]. French-speaking participants were also recruited through community organizations supporting youth and families, in addition to social media (i.e., Facebook groups), word of mouth and the University of Ottawa listserv. To be eligible to participate in this study, adolescents had to be aged between 10 and 17 years, reside in Canada and able to respond to surveys in English or French. Quota sampling was used for gender, where we aimed to recruit at least 8 male participants in each language. Efforts were also made to ensure that adolescents were distributed across each interview round so that younger (10–13 years of age) and older (14–17 years of age) participants were represented in each round of cognitive interviews. We also aimed for diversity in terms of ethnicity among participants. Adolescents and their parents gave their assent to participate, or for their child to participate in the study, respectively. A $25 Amazon gift card was provided to all participants as a thank you for their involvement in this study. The study was approved by the Research Ethics Boards of the University of Guelph (REB# 21-02-010), the University of Ottawa (REB# H-11-23-9832) and Health Canada (REB# 2023- H).

##### Cognitive interview procedures

Cognitive interviews were conducted between February and April 2024. To confirm eligibility and support efforts to engage a diverse sample, parents of interested participants provided the research team with information on their child’s country of residence, language, age, gender and ethnicity by email. In advance of the cognitive interviews, they were emailed an assent form for them and their child to read. The interviews were conducted using Microsoft Teams and were performed by five members of the research team who received training in cognitive interviewing (English, interviewer: MA, note taker: RJ; French, interviewers and note takers: VD, GB and RAL). An iterative process was followed with interviews conducted in rounds. We aimed to include 8 to 10 participants per round in each language as this number of participants is considered sufficient for problematic items to emerge [41, 43]. Items not deemed satisfactory in the initial round were subjected to further testing in subsequent rounds. The number of rounds was not predetermined but we expected that 2 to 3 rounds of interviews would be sufficient to ensure adequate comprehensibility for all items [44–47].

Each cognitive interview began by asking the participant to provide information on their age, gender, ethnicity and ability to respond to survey questions in the language of the interview (English or French). This step was followed by 1) obtaining their verbal assent to participate in the study and to video record the interview, 2) explaining how the interview would be conducted, and 3) performing a practice question with the participant. Using the share screen function, the screener instructions were first shown to the participant, followed by each item with its response scale shown one at the time. After the participant had read a question and provided their response, concurrent verbal probing was used to verify their comprehension, information retrieval, judgement or estimation, and response selection for each item according to Tourangeau’s 4-stage cognitive model of the survey response process [40, 43, 48]. Examples of probing questions were "*In your own words*,* what is this question asking?*", "*Was there anything confusing about this question?"*, *"What does the word [X] mean to you*?", "*What other words would you use instead of [X]*?" (comprehension), "*Was it easy or hard to decide what your answer should be? What made it [easy/hard]?"* (retrieval of information, judgement), "*How sure are you about your answer?"* (judgement), *"Are the response choices clear?*" and *"How would you make the response choices easier to understand*?" (response selection). Each interview lasted approximately 30 min, which also included time to get participant’s feedback on items related to a food literacy screener that was developed as part of this study. The development of this food literacy screener will be reported in a forthcoming publication. Before starting the cognitive interviews with participants, two practice interviews were conducted in each language with adolescents of the targeted age group. Given the practice interviews were successfully conducted with no required changes to the interview scripts, these participants were included in the study sample.

Detailed notes were taken during each cognitive interview. Notes and participant responses were collated in an Excel file to identify common themes for each item to draw conclusions on item comprehensibility and identify any modifications that needed to be made. Participants’ response range was considered to avoid floor or ceiling effects in responses. Following each round of cognitive interviews, the research team met with advisors from Health Canada to discuss the results of the English and French versions of the screener, and provide recommendations, where appropriate, for item wording, response scales, or the screener instructions. Revised items or those whose adequacy was uncertain after the first round were tested in the following round of cognitive interviews. Modifications made in one language were applied to the other language to ensure comparability (i.e., semantic and conceptual equivalence) between the English and French versions of the screener [49, 50]. These items were also retested in the next round of cognitive interviews.

## Results

### Expert panel

Among the 26 items that were evaluated by the 20 members of the expert panel, 11 items did not meet the threshold for expert agreement on relevance or clarity. Among these 11 items, 3 emerged as problematic in both languages, while 6 items were considered problematic in English only and 2 in French only. More specifically, in English, 3 items did not meet expert agreement on relevance and 8 did not meet agreement on clarity. In French, 4 items did not meet expert agreement on relevance and 1 item did not meet agreement on clarity. Most of these items were removed (*n* = 6) or modified (*n* = 4). Among the 4 modified items, 2 items were merged into a single item, resulting in a total of 3 modified items remaining in the screener. Expert comments allowed us to understand why these items were problematic and to refined them so that they were clear and relevant. For some items, the lack of relevance and/or clarity was mainly due to the format in which they were presented to the experts (Excel spreadsheet). This was easily corrected by taking into account expert comments and by modifying the format in which the questionnaire was presented for used with adolescents. One item was also left unchanged. Indeed, despite that the item "*I enjoy eating"* did not quite meet the threshold for relevance in both languages (English = 75%; French = 71%), this item was moved to the stage of cognitive interviewing with adolescents. This decision was motivated by the fact that this item directly assesses the “enjoy your food” recommendation in CFG-2019, and is included in a validated questionnaire that evaluates enjoyment of food and other eating behaviours in youth aged 11–18 years [24, 25], which suggests that this item is appropriate for use with adolescents.

Among the 15 items that met the threshold of more than 75% of experts rating them as relevant and clear in both French and English, 7 items were removed to prioritize those that directly assessed key aspects of CFG-2019 recommendations, and 3 items were modified to simplify the wording. The screener instructions were also slightly modified. In terms of comprehensibility, although some experts suggested additional constructs for the screener, no new items were added, as all key aspects of CFG-2019 recommendations were sufficiently covered while maintaining the screener’s brevity. Following this stage, a total of 12 items were included in the screener that was tested through cognitive interviews with adolescents.

### Cognitive interviews with adolescents

Two rounds of cognitive interviews were sufficient to reach adequate comprehensibility for the screener. A total of 31 adolescents participated in the cognitive interviews (English, *n* = 18; French, *n* = 13) (Table 1). Of them, 17 were included in the first round (English, *n* = 10; French, *n* = 7) and 14 were included in the second round (English, *n* = 8; French, *n* = 6) of cognitive interviews. Fifty-eight percent of participants identified as male and 71.0% were between the ages of 10 and 13 years. Most participants were White, but diverse ethnicities were also represented in the sample. Both rounds of cognitive interviews included younger (10–13 years of age) and older (14–17 years of age) participants. More specifically, the first and second rounds included 13 and 9 participants from the younger age group, respectively.

**Table 1:** Characteristics of adolescents participating in the cognitive interviews

	All (*n* = 31)	English (*n* = 18)	French (*n* = 13)
Gender			
Male	18 (58.1)	10 (55.6)	8 (61.5)
Female	12 (38.7)	7 (38.9)	5 (38.5)
Non-binary	1 (3.2)	1 (5.6)	0 (0)
Age			
10–13 y	22 (71.0)	11 (61.1)	11 (84.6)
14–17 y	9 (29.0)	7 (38.9)	2 (15.4)
Ethnicity			
White	26 (83.9)	13 (72.2)	13 (100)
Other ^1^	5 (16.1)	5 (27.8)	0 (0)

Values are presented as n (%)

^1^ Other includes Black (*n* = 1) and mixed ethnicities (*n* = 4)

Out of the 12 items that were tested in the first round of cognitive interviews, a total of 6 items in English and 5 items in French were retested in the second round of cognitive interviews (Tables 2 and 3). Five items in English (i.e., items 1, 2, 3, 8 and 10) and four items in French (i.e., items 1, 2, 8 and 10) didn’t require any modifications as they were understood as intended, the response options were adequate, and no floor or ceiling effects in responses were observed. Most changes were related to item comprehensibility, while changes made to the screener instructions were related to information retrieval. These changes are detailed below and summarized in Tables 2 and 3.

**Table 2:** Overview of changes by rounds of cognitive interviews for the English version of the Canadian Eating Practices Screener for Adolescents

Original Instructions		Final Instructions		Tested in round 2	Round when revised
The following questions ask about your eating behaviours. For each question, please answer based on what you do in a normal week.		The following questions ask about your eating behaviours. For each question, please answer based on what you do in a normal week **and consider all meals**,** including breakfast**,** lunch and dinner/supper**,** and snacks.**		Yes	Revised after round 1 and after round 2
**Original Item**	**Original Response Scale**	**Final Item**	**Final Response Scale**		
I watch TV or use my mobile phone or tablet during meals.	Never Rarely Sometimes Often Always	1. I watch TV or use my mobile phone or tablet during meals.	Never Rarely Sometimes Often Always	No	-
I take time to eat my meals.	Never Rarely Sometimes Often Always	2. I take time to eat my meals.	Never Rarely Sometimes Often Always	Yes	-
I notice when I am hungry and when I am full.	Never Rarely Sometimes Often Always	3. I notice when I am hungry and when I am full.	Never Rarely Sometimes Often Always	No	-
In a normal week, how often do you help prepare a meal?	Never 1 time per week 2 times per week 3 times per week 4 or more times per week	4. In a normal week, how often do you help **cook** a meal?	Never 1 time per week 2 times per week 3 times per week 4 or more times per week	Yes	Revised after round 1
In a normal week, how often do you help plan your meals?	Never 1 time per week 2 times per week 3 times per week 4 or more times per week	5. In a normal week, how often do you **suggest ideas** for meals?	Never 1 time per week 2 times per week 3 times per week 4 or more times per week	Yes	Revised after round 2^1^
In a normal week, how often do you eat a meal that was prepared at home?	Never 1 time per week 2 times per week 3 times per week 4 or more times per week	6. In a normal week, how often do you eat a **homemade** meal?	**Never** **1–2 days per week** **3–4 days per week** **5–6 days per week** **Everyday**	Yes	Revised after round 1
In a normal week, how often do you eat foods that were prepared outside your home? Include foods from: restaurants, fast food restaurants, convenience stores, and cafeterias.	Never 1–2 days per week 3–4 days per week 5–6 days per week Everyday	7. In a normal week, how often do you eat foods **that come from restaurants**,** fast food restaurants**,** convenience stores**,** and cafeterias?**	Never 1–2 days per week 3–4 days per week 5–6 days per week Everyday	No	Revised after round 2 to march edits to the French version.
I enjoy eating.	Strongly disagree Disagree Neither disagree or agree Agree Strongly agree	8. I enjoy eating.	Strongly disagree Disagree Neither disagree or agree Agree Strongly agree	No	-
I eat with other people when possible.	Never Rarely Sometimes Most days Everyday	9. **When possible**,** I eat with others**,** such as family and friends.**	Never Rarely Sometimes Most days Everyday	Yes	Revised after round 1
I read nutrition information on a food package, such as the nutrition facts table or the ingredient list.	Never Rarely Sometimes Often Always	10. I read nutrition information on a food package, such as the nutrition facts table or the ingredient list.	Never Rarely Sometimes Often Always	No	-
I am aware that food advertising can make me want to eat or drink.	Strongly disagree Disagree Neither disagree or agree Agree Strongly agree	-	-	No	Removed after round 1
Food advertisements can influence what I eat or drink.	Strongly disagree Disagree Neither disagree or agree Agree Strongly agree	11. Food **and drink** advertisements can influence what I eat or drink.	Strongly disagree Disagree Neither disagree or agree Agree Strongly agree	Yes	Revised after round 1

^1^ This item is from a food literacy screener that was tested in round 1 and 2 of cognitive interviews of this study

Bold indicates changes

**Table 3:** Overview of changes by rounds of cognitive interviews for the French version of the Canadian Eating Practices Screener for Adolescents

Original Instructions		Final Instructions		Tested in round 2	Round when revised
Les questions suivantes portent sur ton alimentation. Pour chaque question, essaie de répondre en fonction de ce que tu manges au cours d’une semaine typique.		Les questions suivantes portent sur ton alimentation. Pour chaque question, **répond** en fonction de ce que tu **fais** au cours d’une semaine typique. **Considère tous les repas comme le déjeuner**,** le diner et le souper, et les collations.**		Yes	Revised after round 1 and after round 2
**Original Item**	**Original Response Scale**	**Final Item**	**Final Response Scale**		
Je regarde la télévision, j’utilise mon téléphone ou ma tablette pendant les repas.	Jamais Rarement Parfois Souvent Toujours	1. Je regarde la télévision, j’utilise mon téléphone ou ma tablette pendant les repas.	Jamais Rarement Parfois Souvent Toujours	No	-
Je prends le temps de manger mes repas.	Jamais Rarement Parfois Souvent Toujours	2. Je prends le temps de manger mes repas.	Jamais Rarement Parfois Souvent Toujours	No	-
Je me rends compte lorsque j’ai faim et quand je me sens rassasié.	Jamais Rarement Parfois Souvent Toujours	3. Je me rends compte **quand** j’ai faim et quand je me sens **plein(e).**	Jamais Rarement Parfois Souvent Toujours	Yes	Revised after round 1 and after round 2
Durant une semaine typique, combien de fois est-ce que tu aides à préparer un repas?	Jamais 1 fois par semaine 2 fois par semaine 3 fois par semaine 4 fois ou plus par semaine	4. Durant une semaine typique, combien de fois est-ce que tu aides à **cuisiner** un repas?	Jamais 1 fois par semaine 2 fois par semaine 3 fois par semaine 4 fois ou plus par semaine	Yes	Revised after round 1
Durant une semaine typique, combien de fois est-ce que tu aides à planifier les repas?	Jamais 1 fois par semaine 2 fois par semaine 3 fois par semaine 4 fois ou plus par semaine	5. Durant une semaine typique, combien de fois est-ce que tu **suggères des idées** de repas?	Jamais 1 fois par semaine 2 fois par semaine 3 fois par semaine 4 fois ou plus par semaine	No	Revised after round 2 to match edits to the English version
Dans une semaine typique, combien de fois est-ce que tu manges un repas qui était préparé à la maison?	Jamais 1 fois par semaine 2 fois par semaine 3 fois par semaine 4 fois ou plus par semaine	6. Durant une semaine typique, combien de fois est-ce que tu manges un repas **qui a été cuisiné** à la maison?	**Jamais** **1 à 2 jours par semaine** **3 à 4 jours par semaine** **5 à 6 jours par semaine** **Tous les jours**	Yes	Revised after round 1 and after round 2
Durant une semaine typique, combien de fois t’arrives-t-il de manger de la nourriture qui a été préparée en dehors de la maison. Inclue la nourriture qui a été préparée des restaurants, restaurants de type « fast-food », dépanneurs et cafétéria.	Jamais 1 fois par semaine 2 fois par semaine 3 fois par semaine 4 fois ou plus par semaine	7. Dans une semaine typique, combien de fois **est-ce que tu manges de la nourriture qui vient d’un restaurant**,** d’un restaurant de type « fast-food »**,** d’un dépanneur ou d’une cafétéria?**	**Jamais** **1 à 2 jours par semaine** **3 à 4 jours par semaine** **5 à 6 jours par semaine** **Tous les jours**	Yes	Revised after round 1 to align with the response scale of the English version and after round 2
Manger m’apporte du plaisir.	Fortement en désaccord En désaccord Pas en accord ni en désaccord En accord Fortement en accord	8. Manger m’apporte du plaisir.	Fortement en désaccord En désaccord Pas en accord ni en désaccord En accord Fortement en accord	No	-
Lorsque c’est possible, j’aime prendre mes repas en compagnie de ma famille et/ou mes amis.	Jamais Rarement Parfois Souvent Tous les jours	9. **Quand c’est possible**,** je mange avec d’autres personnes comme ma famille et mes amis.**	Jamais Rarement Parfois **La plupart des jours** Tous les jours	Yes	Revised after round 1, and after round 2 to align with the response scale of the English version
J’utilise l’information disponible sur l’emballage des aliments comme les étiquettes nutritionnelles ou la liste des ingrédients.	Jamais Rarement Parfois Souvent Toujours	10. J’utilise l’information disponible sur l’emballage des aliments comme les étiquettes nutritionnelles ou la liste des ingrédients.	Jamais Rarement Parfois Souvent Toujours	No	-
La publicité alimentaire peut me donner envie de manger ou de boire.	Fortement en désaccord En désaccord Pas en accord ni en désaccord En accord Fortement en accord	-	-	No	Removed after round 1
La publicité alimentaire peut influencer ce que je décide de manger ou de boire.	Fortement en désaccord En désaccord Pas en accord ni en désaccord En accord Fortement en accord	11. La publicité alimentaire peut influencer ce que je **choisis** de manger ou de boire.	Fortement en désaccord En désaccord Pas en accord ni en désaccord En accord Fortement en accord	No	Revised after round 2

Bold indicates changes

Although the screener instructions were clear for participants, a specification to consider all meals and snacks when responding to the screener items was added to the instructions after the initial round. This specification was included to ensure participants considered all of their eating occasions in their responses and to align with the instructions of the adult version of the screener [8, 9]. The revised instructions were well understood by participants from round 2. Minor edits were also made to the English version of the screener instructions after round 2 to improve clarity and alignment with the French version.

For item 3, i.e., “*I notice when I am hungry and when I am full.*” no modifications were required in English. In French, the word “*rassasié*” (i.e., full) was changed for “*plein.e*” after round 1 as it was difficult to understand for some participants. The word “*plein.e*” was clear and well understood by participants from round 2. Minor edits have also been made to improve the syntax of the French version of the item, whose final version reads as “*Je me rends compte quand j’ai faim et quand je me sens plein(e).”*

For item 4, i.e., “*In a normal week*,* how often do you help prepare a meal?”*, several English- and French-speaking participants included setting the table as a way of helping to prepare a meal. To improve comprehension, the item was modified to “*In a normal week*,* how often do you help cook a meal?".* The revised item was clear and understood as intended in round 2.

Although item 5, i.e., “*In a normal week*,* how often do you help plan your meals?”* was clear and well understood in round 1 in both English and French, it was retested in round 2 to determine whether the edited screener instructions would impact participants’ interpretation and responses for this item. In round 2, two English-speaking participants interpreted the item as helping to make a weekly meal schedule specifying which meals would be eaten when rather than simply suggesting ideas for meals. To address this misinterpretation, the item was modified to “*In a normal week*,* how often do you suggest ideas for meals?”*. This item was tested in both rounds of cognitive interviews because it was included in the food literacy screener that was being developed simultaneously. No further testing was required as this item was clear and well understood in both languages.

Item 6, i.e., “*In a normal week*,* how often do you eat a meal that was prepared at home?*" was well understood by most participants, but a few English-speaking participants included store-bought ready-to-eat meals heated at home as meals prepared at home. To avoid this confusion, the item was modified to “*In a normal week*,* how often do you eat a homemade meal?"*. The edited item was clear and well understood in round 2. In addition, the response scale was modified after round 1 due to the observation of a ceiling effect in both English- and French-speaking participants.

Item 7, i.e., " *In a normal week*,* how often do you eat foods that were prepared outside your home? Include foods from: restaurants*,* fast food restaurants*,* convenience stores*,* and cafeterias*.” was clear and well understood in both languages by participants from round 1. However, given that a different response scale was used in French in round 1, the item was retested using the same response scale as the English version in round 2 among French-speaking participants. No issue was observed in round 2. The item has been slightly edited after round 2 in both languages to improve readability so that the final item reads as “*In a normal week*,* how often do you eat foods that come from restaurants*,* fast food restaurants*,* convenience stores*,* and cafeterias?"*.

To reflect the CFG-2019 recommendation to eat meals with others, item 9 tested “*I eat with other people when possible.*” in English and “*Lorsque c’est possible*,* j’aime prendre mes repas en compagnie de ma famille et/ou mes amis. (When possible*,* I enjoy eating my meals with my family and/or friends.)*” in French in round 1. The French item performed well but given that it had a different meaning than the item used in English and that the latter better aligned with the recommendation, the French item was modified to match the item used in English. In round 1, half of the English-speaking participants included only family members in their response and one participant interpreted “others” as individuals outside their family. To avoid this confusion, the item was modified to “*When possible*,* I eat with others*,* such as family and friends.”.* This revised item was clear and well understood in both languages in round 2. In addition, a slight modification was made to the response scale of the French version after round 2 to better align with the English version [i.e., changing *“souvent” (often)* to *“la plupart des jours” (most days)*].

The item “*I am aware that food advertising can make me want to eat or drink.*” was generally well understood, but participants from both language groups found it repetitive compared to the last item of the screener (item 11) and considered the latter clearer. In light of these findings and given the CFG-2019 recommendation “Be aware that food marketing can influence food choices” was covered by item 11, this item was removed from the screener.

Item 11, i.e., “*Food advertisements can influence what I eat or drink.*" , was well understood in both languages by the majority of participants. In the English version, one participant found it unclear to indicate that “food advertisements can influence what I […] drink” and suggested adding “drink” to the noun group of the sentence. The item was modified to “*Food and drink advertisements can influence what I eat or drink.*” and performed well in round 2. In French, the item “*La publicité alimentaire peut influencer ce que je décide de manger ou de boire.”* performed well, although a small edit was made to make the item a little easier to read and improve the translation by making it sound more natural for adolescents, so the final item reads as “*La publicité alimentaire peut influencer ce que je choisis de manger ou de boire."*

With regard to the retrieval of information and response selection, participants generally exhibited no difficulty in providing their answers to the items, did so promptly, and deemed the response options reflected their desired answers. Minor edits were made to some items and the instructions of the French version of the screener to improve the translation and ensure linguistic accuracy. In light of the findings from cognitive interviews, a total of 11 items were retained in the Canadian Eating Practices Screener for Adolescents. The final English and French versions of the screener, the scoring for each item, and the corresponding CFG-2019 recommendation for each item are presented in Table 4 and Supplementary Files S2 and S3. Items 1 and 7 are reverse coded. All items are scored on a scale from 1 to 5, and item scores are summed to yield a total questionnaire score ranging from 11 to 55 points.

**Table 4:** Final version of the Canadian Eating Practices Screener for Adolescents, response options and scoring

	English		French	
**General Instruction**	The following questions ask about your eating behaviours. For each question, please answer based on what you do in a normal week and consider all meals, including breakfast, lunch and dinner/supper, and snacks.		Les questions suivantes portent sur ton alimentation. Pour chaque question, répond en fonction de ce que tu fais au cours d’une semaine typique. Considère tous les repas comme le déjeuner, le diner et le souper, et les collations.	
**CFG-2019 recommendations**	**Item**	**Response options**	**Item**	**Response options**
Be mindful of your eating habits - Take time to eat	1. I watch TV or use my mobile phone or tablet during meals.	o Never (5) o Rarely (4) o Sometimes (3) o Often (2) o Always (1)	1. Je regarde la télévision, j’utilise mon téléphone ou ma tablette pendant les repas.	o Jamais (5) o Rarement (4) o Parfois (3) o Souvent (2) o Toujours (1)
Be mindful of your eating habits - Take time to eat	2. I take time to eat my meals.	o Never (1) o Rarely (2) o Sometimes (3) o Often (4) o Always (5)	2. Je prends le temps de manger mes repas.	o Jamais (1) o Rarement (2) o Parfois (3) o Souvent (4) o Toujours (5)
Be mindful of your eating habits - Notice when you are hungry and when you are full	3. I notice when I am hungry and when I am full.	o Never (1) o Rarely (2) o Sometimes (3) o Often (4) o Always (5)	3. Je me rends compte quand j’ai faim et quand je me sens plein(e).	o Jamais (1) o Rarement (2) o Parfois (3) o Souvent (4) o Toujours (5)
Cook more often	4. In a normal week, how often do you help cook a meal?	o Never (1) o 1 time per week (2) o 2 times per week (3) o 3 times per week (4) o 4 or more times per week (5)	4. Durant une semaine typique, combien de fois est-ce que tu aides à cuisiner un repas?	o Jamais (1) o 1 fois par semaine (2) o 2 fois par semaine (3) o 3 fois par semaine (4) o 4 fois ou plus par semaine (5)
Cook more often - Plan what you eat	5. In a normal week, how often do you suggest ideas for meals?	o Never (1) o 1 time per week (2) o 2 times per week (3) o 3 times per week (4) o 4 or more times per week (5)	5. Durant une semaine typique, combien de fois est-ce que tu suggères des idées de repas?	o Jamais (1) o 1 fois par semaine (2) o 2 fois par semaine (3) o 3 fois par semaine (4) o 4 fois ou plus par semaine (5)
Cook more often	6. In a normal week, how often do you eat a homemade meal?	o Never (1) o 1–2 days per week (2) o 3–4 days per week (3) o 5–6 days per week (4) o Everyday (5)	6. Durant une semaine typique, combien de fois est-ce que tu manges un repas qui a été cuisiné à la maison?	o Jamais (1) o 1 à 2 jours par semaine (2) o 3 à 4 jours par semaine (3) o 5 à 6 jours par semaine (4) o Tous les jours (5)
Cook more often	7. In a normal week, ow often do you eat foods that come from restaurants, fast food restaurants, convenience stores, and cafeterias?	o Never (5) o 1–2 days per week (4) o 3–4 days per week (3) o 5–6 days per week (2) o Everyday (1)	7. Dans une semaine typique, combien de fois est-ce que tu manges de la nourriture qui vient d’un restaurant, d’un restaurant de type « fast-food », d’un dépanneur ou d’une cafétéria?	o Jamais (5) o 1 à 2 jours par semaine (4) o 3 à 4 jours par semaine (3) o 5 à 6 jours par semaine (2) o Tous les jours (1)
Enjoy your food	8. I enjoy eating.	o Strongly disagree (1) o Disagree (2) o Neither disagree or agree (3) o Agree (4) o Strongly agree (5)	8. Manger m’apporte du plaisir.	o Fortement en désaccord (1) o En désaccord (2) o Pas en accord ni en désaccord (3) o En accord (4) o Fortement en accord (5)
Eat meals with others	9. When possible, I eat with others, such as family and friends.	o Never (1) o Rarely (2) o Sometimes (3) o Most days (4) o Everyday (5)	9. Quand c’est possible, je mange avec d’autres personnes comme ma famille et mes amis.	o Jamais (1) o Rarement (2) o Parfois (3) o La plupart des jours (4) o Tous les jours (5)
Use food labels	10. I read nutrition information on a food package, such as the nutrition facts table or the ingredient list.	o Never (1) o Rarely (2) o Sometimes (3) o Often (4) o Always (5)	10. J’utilise l’information disponible sur l’emballage des aliments comme les étiquettes nutritionnelles ou la liste des ingrédients.	o Jamais (1) o Rarement (2) o Parfois (3) o Souvent (4) o Toujours (5)
Be aware that food marketing can influence your choices	11. Food and drink advertisements can influence what I eat or drink.	o Strongly disagree (1) o Disagree (2) o Neither disagree or agree (3) o Agree (4) o Strongly agree (5)	11. La publicité alimentaire peut influencer ce que je choisis de manger ou de boire.	o Fortement en désaccord (1) o En désaccord (2) o Pas en accord ni en désaccord (3) o En accord (4) o Fortement en accord (5)

## Discussion

The Canadian Eating Practices Screener for Adolescents is designed to rapidly assess eating practices based on CFG-2019 recommendations on healthy eating habits among English- and French-speaking adolescents aged 10–17 years living in Canada. The 11-item screener was developed through a comprehensive and iterative process that included the identification of guiding principles, a literature review to inform the adaptation of the adult version of the Canadian Eating Practices Screener for use with adolescents, and the assessment of content validity with experts and through two rounds of cognitive interviews with English- and French-speaking adolescents. The results provide support for content validity of the screener in both languages.

The methodology used to adapt the adult eating practices screener for adolescents enabled us to develop an easy-to-use brief questionnaire whose content validity has been demonstrated by experts and members of the target population. The expert assessment and ongoing input from advisors from Health Canada allowed us to refine the initial items to ensure they were clear and relevant, and to prioritize those that directly assessed CFG-2019 recommendations on healthy eating habits and were the most relevant for adolescents. The cognitive interviews with adolescents led to the deletion of one item that was more difficult to answer and redundant with another item, resulting in an 11-item screener. The final screener includes 3 items assessing eating practices related to mindful eating, 4 items assessing the cook more often recommendation, and one item assessing each of the remaining four eating practices, namely enjoy your food, eat meals with others, use food labels and be aware that food marketing can influence food choices. Given its brevity, the Canadian Eating Practices Screener for Adolescents does not assess all of the eating practices recommended in CFG-2019 [1]. For instance, in contrast to the adult screener [8, 9], the recommendation that cultures and food traditions can be part of healthy eating, which is a subcomponent to the enjoy your food recommendation, is not represented in the adolescent screener.

As demonstrated in prior studies [44, 45, 47], cognitive interviewing with adolescents provided valuable insights that were instrumental in refining the screener and ensuring its clarity and comprehensibility for the target population. The unique perspective of adolescents on their own context revealed issues with some items that had not been anticipated by experts involved in the screener development. For instance, one of the comments made us realize that some participants had a literal understanding of item 11, which prompted the addition of “drink advertisements” to “food advertisements” so that the final item reads as “*Food and drink advertisements can influence what I eat or drink.*”. This observation has also been documented in another study that employed cognitive interviewing to develop a survey for use with adolescents [45]. The comments provided by participants also led to the use of more precise and explicit vocabulary, leaving less room for interpretation. For example, “*help prepare a meal*” was modified to “*help cook a meal*” to ensure that adolescents avoid including setting the table in their responses (item 4). Some comments also led to the use of simpler language and more concise wording. For example, the word “*rassasié*” was changed for “*plein(e)*” in the French version of item 3, while item 7, which originally comprised two sentences, was shortened to a single sentence. These findings resulting in the use of more concise, simple, and explicit vocabulary following cognitive interviews with adolescents have also been observed in other studies [44, 45, 47].

Furthermore, conducting cognitive interviews in two languages concurrently proved beneficial to the development of the screener. In some instances, adolescents provided useful ideas and feedback for rephrasing an item in one language, which, in turn, helped improve the same item in the other language (e.g., item 7). The comments provided by participants were generally very consistent in both languages, suggesting semantic and conceptual equivalence between the two versions of the screener [49, 50].

Overall, the feedback provided by adolescents led to relatively minor adjustments to the screener. However, these modifications had a substantial impact on enhancing its content validity and resulted in a screener whose items are simplified, more explicit, understood as intended, and easy for adolescents to answer. The resulting 11-item Canadian Eating Practices Screener for Adolescents will be useful to assess the overall alignment with CFG-2019 recommendations on healthy eating habits among English- and French-speaking adolescents aged 10–17 years living in Canada. Prior to being used for this purpose, the screener needs to be assessed for construct validity and reliability, including internal consistency and test-retest reliability, among an ethnically diverse sample of English- and French-speaking adolescents aged 10–17 years living in Canada [30]. Future research could also explore whether this screener is responsive to change, which would allow it to be used within longitudinal and intervention studies [51].

Strengths of this study include the use of a robust, comprehensive, and iterative process to adapt the Canadian Eating Practices Screener, which was initially developed for adults aged 18–65 years [8, 9], into a version that is appropriate for use with adolescents. The expert review and cognitive interviewing with adolescents were conducted in both French and English, ensuring the development of a bilingual screener demonstrating content validity in the two official languages of Canada. In terms of limitations, although we sought to ensure age and racial diversity, younger (10–13 years of age) and White participants were overrepresented in the French-speaking sample. However, the results of the cognitive interviews were highly consistent across languages, suggesting that the lack of age and ethnic diversity did not influence the results of the cognitive interviews nor the final version of the French screener. Adolescent participants were mainly recruited in Guelph and the National Capital Region (Ottawa-Gatineau), which may limit the generalizability of results to the entire Canadian population aged 10–17 years. Lastly, although social desirability bias in participants’ responses during cognitive interviews cannot be ruled out, efforts were made to minimize this bias by informing participants that there were no right or wrong answers, and that we were not interested in their actual behaviours, but rather in what they think about when answering each question and whether the questions are clear.

## Conclusions

The Canadian Eating Practices Screener for Adolescents/Questionnaire court canadien sur les pratiques alimentaires des adolescents was designed to rapidly assess eating practices based on CFG-2019 recommendations on healthy eating habits among English- and French-speaking adolescents aged 10–17 years living in Canada. The comprehensive and bilingual development process which included content validity assessment by experts and comprehensibility assessment through cognitive interviewing with adolescents resulted in an 11-item screener that demonstrates a high level of content validity in both English and French. After being further tested for construct validity, internal consistency, test-retest reliability and responsiveness to change, this measure will be useful for research purposes and for the monitoring and surveillance of eating practices among adolescents living in Canada.

## Supplementary Information

Supplementary material 1.

## Data Availability

Data are available from the corresponding author upon reasonable request. The questionnaire is available in English and French as supplementary files.

## Abbreviations

CFG: Canada's Food Guide
COSMIN: COnsensus-based Standards for the selection of health Measurement INstruments
