# Supplementary material for: Development of the Canadian Eating Practices Screener for Adolescents to assess eating practices based on Canada’s Food Guide 2019 recommendations
Source: Int J Behav Nutr Phys Act. 2025 Dec 16;23:6. doi: 10.1186/s12966-025-01853-1 (PMC12822044; doi:10.1186/s12966-025-01853-1)
Supplement: Supplementary file 1 — Supplementary material 1. [file 12966_2025_1853_MOESM1_ESM.docx]

**Supplementary files**

Development of the Canadian Eating Practices Screener for Adolescents to assess eating practices based on Canada’s Food Guide 2019 recommendations

Jacob R et al.

Online Supplemental Material

Supplementary File S1. Advisors who provided input on the development of the Canadian Eating Practices Screener for Adolescents

**French:**

Véronique Gingras, Université de Montréal, Canada

Raphaëlle Jacob, University of Guelph, Canada

Jacynthe Lafrenière, Health Canada

Simone Lemieux, Université Laval, Canada

Isabelle Massarelli, Health Canada

Maude Perreault, Université de Montréal, Canada

Véronique Provencher, Université Laval, Canada

**English:**

Kate Bauer, University of Michigan, USA

Heidi Boyd, Government of Newfoundland and Labrador, Canada

Tina Busetto, Government of Yukon, Canada

Shawn Brulé, Statistics Canada

Lisa-Anne Elvidge Munene, Health Canada

Katie Loth, University of Minnesota, USA

Alicia E. Martin, University of Guelph, Canada

Isabelle Massarelli, Health Canada

Shannon Olsen, Health Canada

Janis Randall Simpson, University of Guelph, Canada

Joyce Slater, University of Manitoba, Canada

Kathryn Walton, University of Guelph, Canada

Supplemental Table 1. Concordance among items from the adolescent and adult versions of the Canadian Eating Practices Screener

|  | **Canadian Eating Practices Screener**  **for Adolescents** | | **Corresponding items from the Canadian Eating Practices Screener developed for adults** ^1^ | |
| --- | --- | --- | --- | --- |
| **Instructions** | The following questions ask about your eating behaviours. For each question, please answer based on what you do in a normal week and consider all meals, including breakfast, lunch and dinner/supper, and snacks. | | The following questions ask about your eating behaviours in the past month. When answering, please consider all meals such as, breakfast, lunch, dinner/supper, and snacks. | |
| **CFG-2019 recommendations** | **Item** | **Response options** | **Item** | **Response options** |
| Be mindful of your eating habits - Take time to eat | I watch TV or use my mobile phone or tablet during meals. | Never  Rarely  Sometimes  Often  Always | I engage in other activities while eating. Examples are working, studying, driving, watching tv, using a computer, phone, or tablet. | Never  Rarely  Sometimes  Often  Always |
| Be mindful of your eating habits - Take time to eat | I take time to eat my meals. | Never  Rarely  Sometimes  Often  Always | I take time to eat my meals. | Never  Rarely  Sometimes  Often  Always |
| Be mindful of your eating habits - Notice when you are hungry and when you are full | I notice when I am hungry and when I am full. | Never  Rarely  Sometimes  Often  Always | 1. I rely on my hunger signals to tell me when to eat. 2. I rely on my fullness signals to tell me when to stop eating. | Never  Rarely  Sometimes  Often  Always |
| Cook more often | In a normal week, how often do you help cook a meal? | Never  1 time per week  2 times per week  3 times per week  4 or more times per week | I cook meals from scratch. This means meals that include at least three basic ingredients. Examples of basic ingredients include canned beans, meats, vegetables, rice, spices, etc. | Never  1-3 times/month  1-3 times/week  4-6 times/week  7 or more times/week |
| Cook more often - Plan what you eat | In a normal week, how often do you suggest ideas for meals? | Never  1 time per week  2 times per week  3 times per week  4 or more times per week | I plan meals ahead of time. | Never  Rarely  Sometimes  Often  Always |
| Cook more often | In a normal week, how often do you eat a homemade meal? | Never  1-2 times per week  3-4 times per week  5-6 times per week  Everyday | I eat meals that are cooked at home from scratch. This means meals that include at least three basic ingredients. Examples of basic ingredients include canned beans, meats, vegetables, rice, spices, etc. | Never  1-3 times/month  1-3 times/week  4-6 times/week  7 or more times/week |
| Cook more often | How often do you eat foods that come from restaurants, fast food places, convenience stores, and cafeterias? | Never  1-2 days per week  3-4 days per week  5-6 days per week  Everyday | I eat food from restaurants. This includes dine-in restaurants, take-out, delivery, or fast food. | Never  1-3 times/month  1-3 times/week  4-6 times/week  7 or more times/week |
| Enjoy your food | I enjoy eating. | Strongly disagree  Disagree  Neither disagree or agree  Agree  Strongly agree | Eating is a pleasure for me. | Strongly disagree  Disagree  Neither disagree or agree  Agree  Strongly agree |
| Eat meals with others | When possible, I eat with others, such as family and friends. | Never  Rarely  Sometimes  Most days  Everyday | When I have the opportunity, I eat my meals with others. | Never  Rarely  Sometimes  Often  Always |
| Use food labels | I read nutrition information on a food package, such as the nutrition facts table or the ingredient list. | Never  Rarely  Sometimes  Often  Always | I use nutrition information on food labels when buying foods and/or drinks for the first time. | Never  Rarely  Sometimes  Often  Always |
| Be aware that food marketing can influence your choices | Food and drink advertisements can influence what I eat or drink. | Strongly disagree  Disagree  Neither disagree or agree  Agree  Strongly agree | I am aware that food advertisements can make me want to eat or drink. | Strongly disagree  Disagree  Neither disagree or agree  Agree  Strongly agree |

^1^ The Canadian Eating Practices Screener validated among adults comprises a total of 21 items (8, 9).

Supplementary File S2. Canadian Eating Practices Screener for Adolescents

The following questions ask about your eating behaviours. For each question, please answer based on what you do in a normal week and consider all meals, including breakfast, lunch and dinner/supper, and snacks.

1. I watch TV or use my mobile phone or tablet during meals.

- Never
- Rarely
- Sometimes
- Often
- Always

1. I take time to eat my meals.

- Never
- Rarely
- Sometimes
- Often
- Always

1. I notice when I am hungry and when I am full.

- Never
- Rarely
- Sometimes
- Often
- Always

1. In a normal week, how often do you help cook a meal?

- Never
- 1 time per week
- 2 times per week
- 3 times per week
- 4 or more times per week

1. In a normal week, how often do you suggest ideas for meals?

- Never
- 1 time per week
- 2 times per week
- 3 times per week
- 4 or more times per week

1. In a normal week, how often do you eat a homemade meal?

- Never
- 1-2 days per week
- 3-4 days per week
- 5-6 days per week
- Everyday

1. In a normal week, how often do you eat foods that come from restaurants, fast food restaurants, convenience stores, and cafeterias?

- Never
- 1-2 days per week
- 3-4 days per week
- 5-6 days per week
- Everyday

1. I enjoy eating.

- Strongly disagree
- Disagree
- Neither disagree or agree
- Agree
- Strongly agree

1. When possible, I eat with others, such as family and friends.

- Never
- Rarely
- Sometimes
- Most days
- Everyday

1. I read nutrition information on a food package, such as the nutrition facts table or the ingredient list.

- Never
- Rarely
- Sometimes
- Often
- Always

1. Food and drink advertisements can influence what I eat or drink.

- Strongly disagree
- Disagree
- Neither disagree or agree
- Agree
- Strongly agree

Supplementary File S3. Questionnaire court canadien sur les pratiques alimentaires des adolescents

Les questions suivantes portent sur ton alimentation. Pour chaque question, répond en fonction de ce que tu fais au cours d'une semaine typique. Considère tous les repas comme le déjeuner, le diner et le souper, et les collations.

1. Je regarde la télévision, j'utilise mon téléphone ou ma tablette pendant les repas.

- Jamais
- Rarement
- Parfois
- Souvent
- Toujours

1. Je prends le temps de manger mes repas.

- Jamais
- Rarement
- Parfois
- Souvent
- Toujours

1. Je me rends compte quand j’ai faim et quand je me sens plein(e).

- Jamais
- Rarement
- Parfois
- Souvent
- Toujours

1. Durant une semaine typique, combien de fois est-ce que tu aides à cuisiner un repas?

- Jamais
- 1 fois par semaine
- 2 fois par semaine
- 3 fois par semaine
- 4 fois ou plus par semaine

1. Durant une semaine typique, combien de fois est-ce que tu suggères des idées de repas?

- Jamais
- 1 fois par semaine
- 2 fois par semaine
- 3 fois par semaine
- 4 fois ou plus par semaine

1. Durant une semaine typique, combien de fois est-ce que tu manges un repas qui a été cuisiné à la maison?

- Jamais
- 1 à 2 jours par semaine
- 3 à 4 jours par semaine
- 5 à 6 jours par semaine
- Tous les jours

1. Durant une semaine typique, combien de fois est-ce que tu manges de la nourriture qui vient d’un restaurant, d'un restaurant de type « fast-food », d'un dépanneur ou d'une cafétéria?

- Jamais
- 1 à 2 jours par semaine
- 3 à 4 jours par semaine
- 5 à 6 jours par semaine
- Tous les jours

1. Manger m’apporte du plaisir.

- Fortement en désaccord
- En désaccord
- Pas en accord ni en désaccord
- En accord
- Fortement en accord

1. Quand c'est possible, je mange avec d’autres personnes comme ma famille et mes amis.

- Jamais
- Rarement
- Parfois
- La plupart des jours
- Tous les jours

1. J'utilise l'information disponible sur l’emballage des aliments comme les étiquettes nutritionnelles ou la liste des ingrédients.

- Jamais
- Rarement
- Parfois
- Souvent
- Toujours

1. La publicité alimentaire peut influencer ce que je choisis de manger ou de boire.

- Fortement en désaccord
- En désaccord
- Pas en accord ni en désaccord
- En accord
- Fortement en accord
